# Spatial memory impairment by TRPC1 depletion is ameliorated by environmental enrichment

**DOI:** 10.18632/oncotarget.8428

**Published:** 2016-03-28

**Authors:** Renzhong Xing, Yanling Zhang, Hua Xu, Xiaobin Luo, Raymond Chuen-Chung Chang, Jianjun Liu, Xifei Yang

**Affiliations:** ^1^ College of Pharmacy, Jinan University, Guangdong, China; ^2^ Key Laboratory of Modern Toxicology of Shenzhen, Medical Key Laboratory of Guangdong Province, Medical Key Laboratory of Health Toxicology of Shenzhen, Shenzhen Center for Disease Control and Prevention, Shenzhen, China; ^3^ Laboratory of Neurodegenerative Diseases, Department of Anatomy, The University of Hong Kong, Pokfulam, Hong Kong SAR, China; ^4^ AND Biotech, Shenzhen, China; ^5^ Guang Zhou Kai-Tuo Biotech, Guangzhou, China

**Keywords:** TRPC1, memory, environment enrichment, α-internexin, GMF-β

## Abstract

Canonical transient receptor potential (TRPC) channels are widely expressed throughout the nervous system whereas their functions remain largely unclear. Here we investigated the effects of TRPC1 deletion on spatial memory ability of mice and the potential intervention by environmental enrichment (EE). Significant spatial memory impairment assessed by conditional fearing test, Y maze test and step-down test in TRPC1 knockout mice was revealed. The behavioral abnormality were attenuated by the treatment of EE. Proteomic analysis by two-dimensional fluorescence difference gel electrophoresis (2D-DIGE) coupled with a matrix-assisted laser desorption/ionisation-time of flight (MALDI-TOF) and tandem mass spectrometry (MS) revealed that TRPC1 deletion caused differential expression of a total of 10 proteins (8 up-regulated and 2 down-regulated) in hippocampus. EE treatment resulted in differential expression of a total of 22 proteins (2 up-regulated and 20 down-regulated) in hippocampus of TRPC1 knockout mice. Among these differentially expressed proteins, the expression of α-internexin and glia maturation factor β (GMF-β), two proteins shown to impair memory, were significantly down-regulated in hippocampus of TRPC1 knockout mice by EE treatment. Taken together, these data suggested that TRPC1 regulated directly or indirectly the expression of multiple proteins, which may be crucial for the maintenance of memory ability, and that EE treatment modulated spatial memory impairment caused by TRPC1 depletion and the mechanisms may involve the modulation of EE on the expression of those dys-regulated proteins such as α-internexin and GMF-β in hippocampus.

## INTRODUCTION

The transient receptor potential (TRP) superfamily of ion channels represents a group of structurally and evolutionarily related cation channels composed of several groups that include the TRPC (canonical), TRPV (vanilloid), TRPM (melastatin), TRPP (polycystin), TRPML (mucolipin), and the TRPA (ankyrin) [1]. The TRPC1 cation channel was the first mammalian TRP channel that was cloned.

The essential role of TRPC1 is its involvement in regulating Ca^2+^ homeostasis. Activation of the G-protein-coupled receptor (GPCR) in the plasma membrane (PM) results in the activation of phospholipase C (PLC) and production of inositol 1, 4, 5-trisphosphate (IP3) from phosphatidylinositol-4,5-bisphosphate (PIP2) [2]. IP3 binds to the IP3 receptor (IP3R) on the endoplasmic reticulum (ER) to induce a rapid Ca^2+^ release from the ER [3]. Intracellular Ca^2+^ is released to extracellular space by plasma membrane Ca^2+^-ATPases and Na^+^–Ca^2+^ exchangers [4]. The depletion of the internal Ca^2+^ stores in the ER causes activation of store-operated Ca^2+^ (SOC) entry channels [5]. SOC-mediated Ca^2+^ entry, a critical process to maintain ER Ca^2+^ levels, is dependent on TRPC1. After store depletion, TRPC1 interaction with the SOCE modulator stromal interaction molecule 1 (STIM1) causes Ca^2+^ influx [5]. TRPC1 knockdown induced cell cycle arrest in G0/G1, indicating that SOCE represents a principal mechanism regulating the proliferation of adult neural progenitor cell (aNPCs) [6]. Ca^2+^ entry via TRPC1 activated the AKT pathway, which has a key role in neuroprotection [7]. TRPC1 knockout mice (TRPC1^−/−^) showed an increased unfolded protein response and loss of dopaminergic neurons in the substantia nigra, a condition closely resembling Parkinson's disease [7]. Neurons obtained from transgenic drosophila models of Huntington's disease (HD) displayed increased store-operated Ca^2+^ entry, which was suppressed by a class of quinazoline-derived compounds. These compounds improved the neurodegenerative phenotype of HD flies and the effects required the expression of TRPC1 [8]. These data suggested the physiological and pathogenic roles of TRPC1 in the nerve system.

Studies have shown that TRPC1 is widely expressed in the ER [9], neuronal dendrites [10], and plasma membrane [11]. The expression of TRPC1 protein was down-regulated in aorta in 16- to 20-month-old rats compared to that from 2-to-4 month-old [12]. The high expression of TRPC1 channels in mammalian temporal lobe structure suggests that TRPC1 may be involved in neuronal plasticity, learning and memory, and also contribute to neuronal survival [7, 13]. Although TRPC1 is highly expressed in hippocampus, whether it is involved in hippocampus-dependent memory remains still elusive.

Environmental enrichment (EE) refers to housing conditions, where animals could strengthen the senses, cognition and movement of the stimulus compared to a normal cage environment [14, 15]. EE involves a combination of increased social interaction, physical exercise and continuous exposure to learning tasks, and can increase levels of neurotrophins, such as nerve growth factor (NGF), which plays integral roles in neuronal signaling [16, 17]. EE was shown to protect against HD, Alzheimer's disease, Down syndrome and stroke [18–21]. EE rescued the loss of proteins, such as brain-derived neurotrophic factor, dopamine- and cAMP-regulated phosphoprotein and cannabinoid receptor 1 in HD [22, 23]. EE was also shown to delay the loss of peristriatal cerebral volume in R6/2 HD mice [24]. In addition, EE was able to decrease Aβ levels and amyloid deposits [25], and increased the expression of BDNF, NGF-A and NGF-B as well as enhanced dendritic growth in stroke [26–28]. These data revealed wide neuroprotective effects via targeting multiple critical pathways or molecules in neurological diseases. In the present study, we are aimed to explore the effects of TRPC1 depletion on spatial memory, and the potential invention of EE treatment and the molecular mechanisms.

## RESULTS

### Spatial memory deficit was caused by TRPC1 depletion and rescued by EE treatment

**Figure 1:**
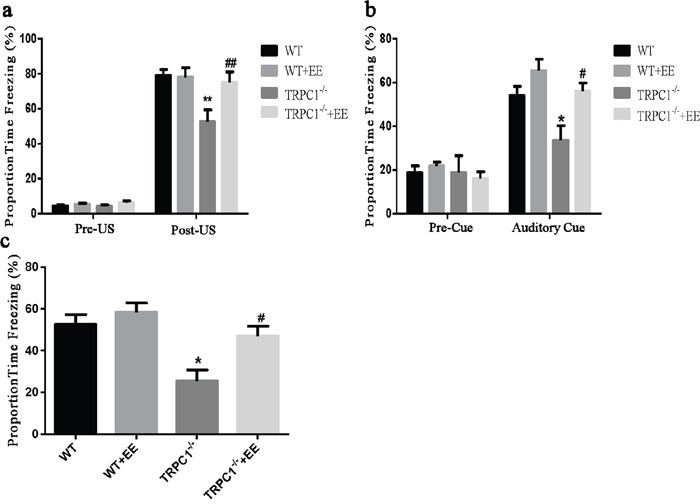
Spatial memory ability was measured by trace cued and contextual fear conditioning **a.** Proportion time freezing during the first 3 min of training before CS-US presentation (Pre-US), and the final 2 min of the training session after CS-US presentations (Post-US). **b.** Freezing behavior in the novel context, 24 h after training during the first 2 min of no CS presentation (Pre-Cue) and 1 min of CS presentation (Auditory Cue). **c.** Freezing behavior 48 h after training, in the same context in which training was carried out on day 1. Values were expressed as mean +/− SEM.^*^, ^**^ *P* < 0.05 and *P* < 0.01, vs. WT, respectively; ^#^, ^##^*P* < 0.05 and *P* < 0.01, vs. TRPC1^−/−^, respectively. *n*=8-13 for each group.

Trace cued and contextual fear conditioning: As shown in Figure 1a, there were no significant differences among the four groups during the first 3 min in the training session before the CS-US presentation. A significant difference in the proportion of time spent on freezing for the last 2 min of the training session was observed (*P* < 0.01). TRPC1^−/−^ mice froze significantly less than the WT mice (*P* < 0.01), indicating that TRPC1 depletion caused spatial memory deficit. TRPC1^−/−^ mice treated with EE froze significantly more than TRPC1^−/−^ mice (*P* < 0.01), indicating that memory deficit was rescued by EE treatment. There were no significant differences among the groups during the first 2 min in the novel context before the CS presentation. TRPC1^−/−^ mice froze significantly less than the WT mice (*P* < 0.05), and TRPC1^−/−^ mice treated with EE froze significantly more than TRPC1^−/−^ mice (*P* < 0.05) during the 60s auditory cue (Figure 1b).

Lastly, when the mice were returned to the training environment for the contextual fear test, a significant difference in the proportion of time spent on freezing among the groups was observed 48h after the training. TRPC1^−/−^ mice froze significantly less than the WT mice (*P* < 0.05), and TRPC1^−/−^ mice treated with EE froze significantly more than the TRPC1^−/−^ mice (*P* < 0.05) (Figure 1c). These data indicated that the memory ability of TRPC1^−/−^ mice was significantly decreased compared to WT mice. The memory ability was significantly enhanced in TRPC1^−/−^ mice after treatment of EE.

**Figure 2:**
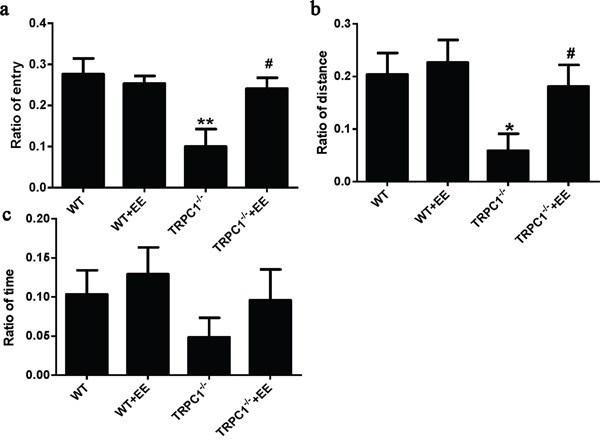
Spatial memory performance in the Y maze **a.** Ratio of entry, **b.** Ratio of distance and **c.** Ratio of time. Values were expressed as mean +/− SEM. ^*^, ^**^ *P* < 0.05 and *P* < 0.01 vs. WT mice, respectively; # *P* < 0.05 vs. TRPC1^−/−^ mice. *n* = 8-10 for each group.

Y maze test: TRPC1^−/−^ mice showed significantly decreased ratio of entry compared with the WT mice (*P* < 0.01) (Figure 2a). TRPC1^−/−^ mice treated with EE displayed higher ratio of entry than TRPC1^−/−^ mice (*P* < 0.05) (Figure 2a). Compared with the WT mice, the ratio of distance for TRPC1^−/−^ mice was significantly decreased (*P* < 0.05) (Figure 2b). However, the ratio of distance was significantly increased in TRPC1^−/−^ mice treated with EE compared with non-treated TRPC1^−/−^ mice (*P* < 0.05) (Figure 2b). There were no significant differences in the ratio of time among all the groups (Figure 2c). These data suggested that TRPC1 depletion impaired the memory of mice and EE treatment could rescue the impairment.

**Figure 3:**
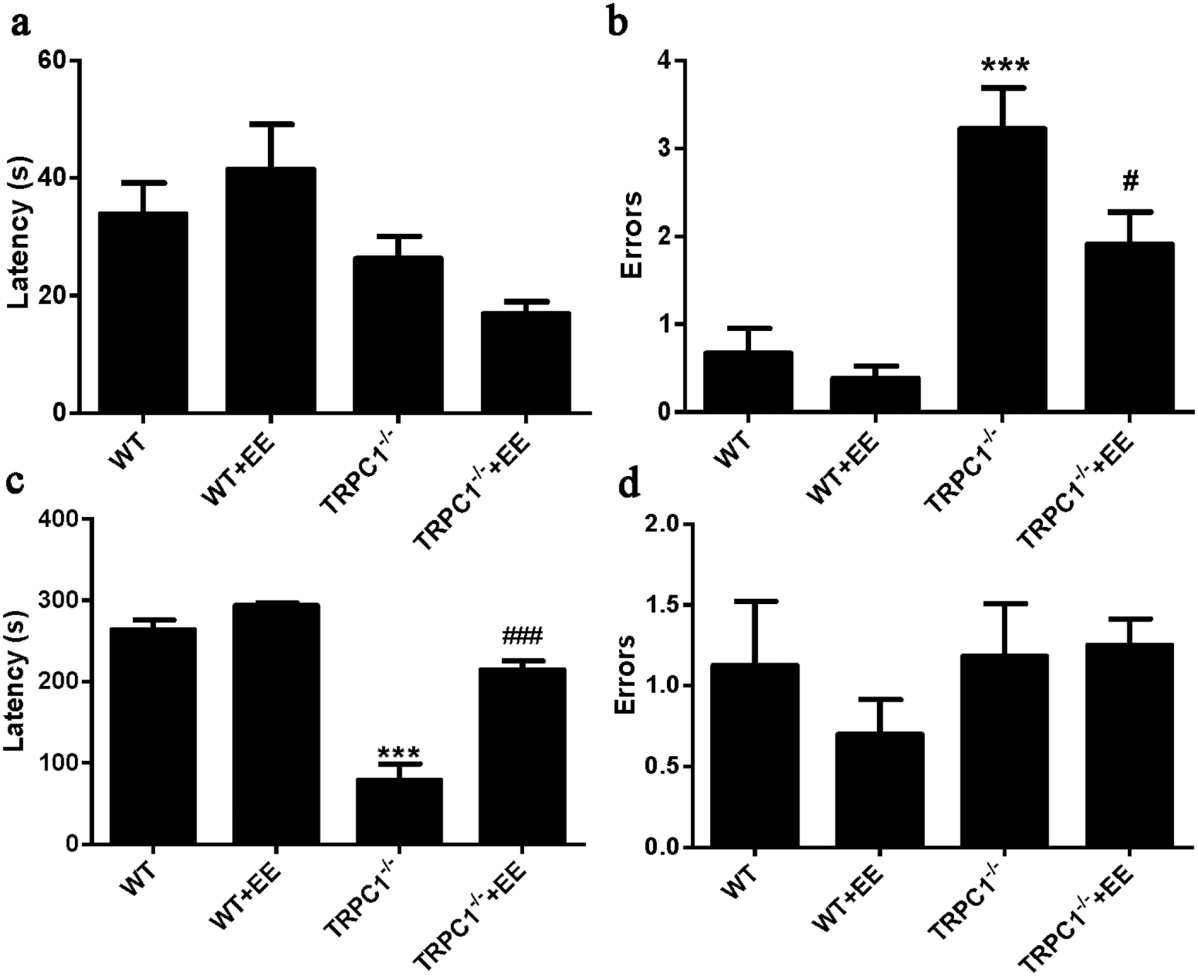
Spatial memory ability was measured by step-down test **a.** Latencies on Day 1, **b.** The number of errors on Day 1, **c.** Latencies on Day 2, **d.** The number of errors on Day 2. Values were represented as mean +/− SEM. ^*^ and ^***^P < 0.05 and *P* < 0.001 vs. WT mice, respectively; ^#^ and ^###^ *P* < 0.05 and *P* < 0.001 vs. TRPC1^−/−^ mice, respectively. *n*=8-10 for each group.

Step-down test: Day 1 was the memory acquisition period. No significant differences in latency were observed among the groups during day 1 (Figure 3a). The number of errors was significantly higher in TRPC1^−/−^ mice compared to the WT mice (*P* < 0.001), and the number of errors was significantly decreased in EE-treated TRPC1^−/−^ mice compared to non-treated TRPC1^−/−^ mice (*P* < 0.05) (Figure 3b). These data indicated that TRPC1 depletion impaired memory acquisition, and EE treatment could prevent the impairment. On Day 2, memory consolidation was evaluated. The latency was significantly shorter in TRPC1^−/−^ mice compared to the WT mice (*P* < 0.001), and the latency was significantly prolonged in EE-treated TRPC1^−/−^ mice compared with non-treated TRPC1^−/−^ mice (*P* < 0.001) (Figure 3c). No significant differences in the number of errors were observed among the groups during the memory consolidation test (Figure 3d). These data further suggested that TRPC1 depletion impaired the memory of mice and EE treatment could rescue the impairment.

### Identification of differentially expressed proteins in hippocampus in WT mice and TRPC1^−/−^ mice treated with or without EE

**Figure 4:**
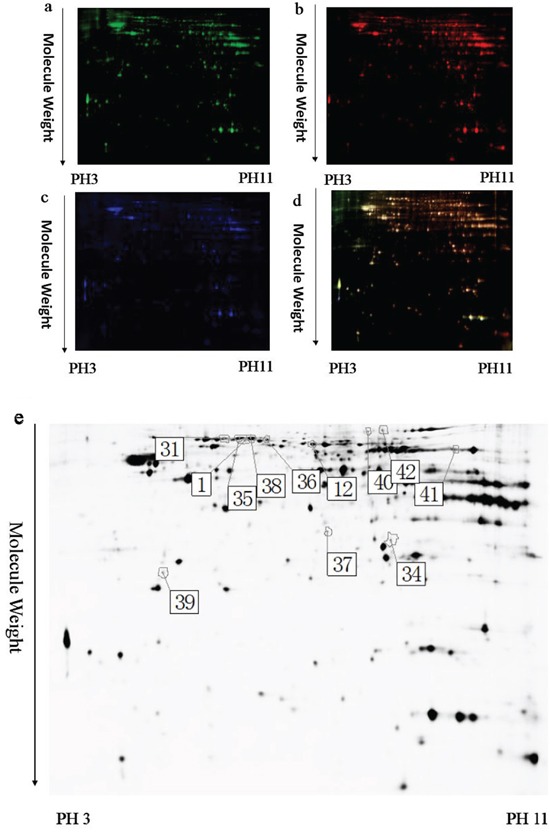
A representative 2D-DIGE gel image of hippocampal proteins from WT mice and TRPC1^−/−^ mice Hippocampal proteins from WT mice and TRPC1^−/−^ mice were labeled with Cy3 or Cy5 dye, respectively (*n* =6 for each group). An internal standard protein sample (a mixture of all hippocampus samples) was labeled with the Cy2 dye. The CyDye-labeled samples were combined, and the proteins were co-separated in the first dimension via IEF in 24 cm pH 3–11 nonlinear IPG strips, followed by separation in the second dimension via SDS-PAGE. Spots of interest were manually excised, digested and subjected to identification by MALDI-TOF-MS/MS. **a.** A representative image of hippocampus proteins from TRPC1^−/−^ mice labeled with Cy5 dye. **b.** A representative image of a 2D-DIGE gel showing Cy3-labeled hippocampus proteins from WT mice. **c.** A representative image of a 2D-DIGE gel showing Cy2-labeled proteins as internal standards. **d.** A merged image of the 2D-DIGE gel displaying Cy2-, Cy3- and Cy5-labeled proteins. **e.** Greyscale 2D-DIGE gel image showing 10 differentially expressed protein spots identified by MALDI-TOF-MS/MS (black numbers with white square) in hippocampus of TRPC1^−/−^ mice relative to WT mice.

**Figure 5:**
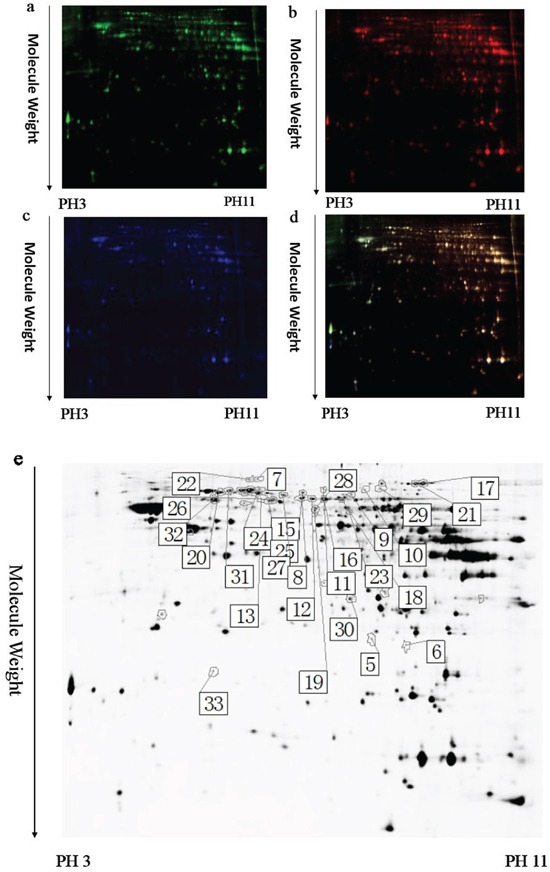
A representative 2D-DIGE gel image of hippocampal proteins from TRPC1^−/−^ mice treated with or without EE Hippocampal proteins from TRPC1^−/−^ treated with or without EE were labeled with Cy3 or Cy5 dye, respectively (*n* =6 for each group). An internal standard protein sample (a mixture of all hippocampus samples) was labeled with the Cy2 dye. The CyDye-labeled samples were combined, and the proteins were co-separated in the first dimension via IEF in 24 cm pH 3–11 nonlinear IPG strips, followed by separation in the second dimension via SDS-PAGE. Spots of interest were manually excised, digested and subjected to identification by MALDI-TOF-MS/MS. **a.** A representative image of hippocampus proteins from EE-treated TRPC1^−/−^ mice labeled with Cy5 dye. **b.** A representative image of a 2D-DIGE gel showing Cy3-labeled hippocampus proteins from TRPC1^−/−^ mice. **c.** A representative image of a 2D-DIGE gel showing Cy2-labeled proteins as internal standards. **d.** A merged image of the 2D-DIGE gel displaying Cy2-, Cy3- and Cy5-labeled proteins. **e.** Greyscale 2D-DIGE gel image showing 22 differentially expressed protein spots identified by MALDI-TOF-MS/MS (black numbers with white square) in hippocampus of EE-treated TRPC1^−/−^ mice relative to non-treated TRPC1^−/−^ mice.

**Figure 6:**
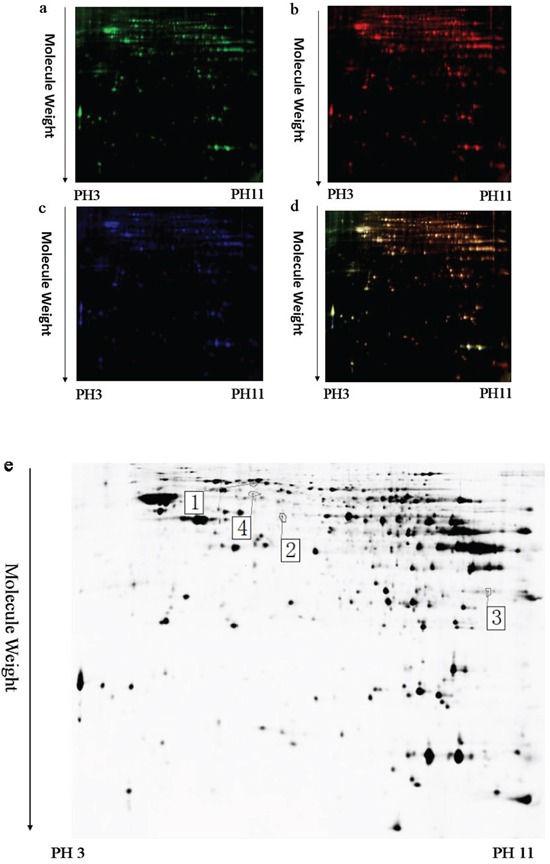
A representative 2D-DIGE gel image of hippocampal proteins of EE-treated WT mice and WT mice The hippocampal samples from EE-treated WT mice and WT mice (*n* = 6 for each group) were labeled with Cy-Dye, multiplexed, and underwent isoelectric focusing on 24 cm pH 3-11 nonlinear IPG strips. The proteins were subsequently separated on large-format 12.5% gels. Spots of interest were manually excised, digested and subjected to identification by MALDI-TOF-MS/MS. **a.** Cy3-labled hippocampus proteins of EE-treated WT mice. **b.** Cy5-labeled hippocampus proteins of WT mice. **c.** Cy2-labeled proteins as internal standards. **d.** The merged image showing Cy2-, Cy3-, and Cy5-labeled proteins. **e.** Greyscale 2D-DIGE gel image showing 3 differentially expressed protein spots identified by MALDI-TOF-MS/MS (black numbers with white square) in hippocampus of EE-treated WT mice relative to WT mice.

To identify the potential molecules involved in memory impairment caused by TRPC1 depletion, comparative proteomic analysis was performed on the brain samples from WT mice and TRPC1^−/−^ mice treated with or without EE. Representative 2D-DIGE gel images of sample proteins acquired from the WT and TRPC1^−/−^ mice treated with or without EE were shown in Figure 4a–4d, 5a–5d and 6a–6d. The spots showing a fold-change of 1.1 or greater and a *P*-value ≤ 0.05 were considered differentially expressed protein spots. Differentially expressed proteins were identified and annotated (Figure 4e, 5e and 6e) in the form of protein ID. The fold-change levels and *p*-values calculated using the DeCyder software were shown in Table 1, Table 2 and Table 3, respectively. Based on the data obtained from the SwissProt database, the gI accession number, the percentage of sequence coverage, the theoretical pI, the Mascot scores, and the theoretical molecular weight of these proteins were also shown in the Table 1, Table 2 and Table 3, respectively. The proteins with at least two different peptide sequences and multiple peptide hits corresponding to every MS/MS event were identified. The MS/MS spectra for two proteins implicated in memory function, α-internexin and GMF-β, were shown in Supplementary Figures 1 and 2.

**Table 1:** Differentially expressed protein spots in hippocampus between TRPC1^−/−^ and WT mice identified via 2D-DIGE/MALDI-TOF-MS/MS

Spot No	Protein ID. ^a^	Accession No.	Protein name ^b^	Mascot score	Cove. ^c^ (%)	MW^d^ (Da)	PI ^e^	TRPC1^−/−^ vs WT	
								Ratio ^f^	*P*-value
434	34	PGAM1_MOUSE	Phosphoglycerate mutase 1	445	18	28928	6.67	1.68	0.003
68	35	ALBU_MOUSE	Serum albumin	528	13	70700	5.75	1.19	0.009
85	36	DPYL2_MOUSE	Dihydropyrimidinase-related protein 2	482	13	62638	5.95	1.34	0.01
427	37	VDAC2_MOUSE	Voltage-dependent anion-selective channel protein 2	41	9	32340	7.44	1.12	0.011
69	38	ALBU_MOUSE	Serum albumin	539	14	70700	5.75	1.36	0.017
74	31	VATA_MOUSE	V-type proton ATPase catalytic subunit A	345	11	68625	5.42	1.14	0.017
489	39	HEBP1_MOUSE	Heme-binding protein 1	59	10	21167	5.14	−1.95	0.017
141	41	ATPA_MOUSE	ATP synthase subunit alpha, mitochondrial	586	13	59830	9.22	1.16	0.034
93	1	ODP2_MOUSE	Dihydrolipoyllysine-residue acetyltransferase component of pyruvate dehydrogenase complex, mitochondrial	383	9	68469	8.81	−1.08	0.038
33	42	FUBP2_MOUSE	Far upstream element-binding protein 2	206	7	77184	6.9	1.28	0.045

a Protein ID assigned manually.

b Protein name identified by MS.

c Sequence coverage achieved by MALDI-TOF-MS.

d Theoretical molecular weight of the protein(s).

e Theoretical isoelectrical point of the protein(s).

f The ratio in spot density from TRPC1^−/−^ mice compared to WT mice. *n* = 6 for both group.

**Table 2:** Differentially expressed protein spots in hippocampus between EE-treated TRPC1^−/−^ mice and non-treated TRPC1^−/−^ mice identified via 2D-DIGE/MALDI-TOF-MS/MS

Spot No	Protein ID. ^a^	Accession No.	Protein name ^b^	Mascot score	Cove. ^c^ (%)	MW^d^ (Da)	PI ^e^	TRPC1^−/−^+EE vs TRPC1^−/−^	
								Ratio^f^	*P*-value
513	5	G3P_MOUSE	Glyceraldehyde-3-phosphate dehydrogenase	117	4	36072	8.44	1.54	0.0056
514	6	G3P_MOUSE	Glyceraldehyde-3-phosphate dehydrogenase	120	4	36072	8.44	1.64	0.0065
20	7	ATPB_MOUSE	ATP synthase subunit beta, mitochondrial	254	10	56265	5.19	−1.5	0.0074
106	8	DPYL2_MOUSE	Dihydropyrimidinase-related protein 2	694	17	62638	5.95	−1.1	0.0089
61	9	NSF_MOUSE	Vesicle-fusing ATPase	170	6	83131	6.52	−1.3	0.0091
70	10	ACTBL_MOUSE	Beta-actin-like protein 2	74	10	42319	5.3	−1.2	0.0092
59	11	DPYL2_MOUSE	Dihydropyrimidinase-related protein 2	486	12	62638	5.95	−1.2	0.015
124	14	AINX_MOUSE	Alpha-internexin	91	12	55879	5.23	−1.3	0.017
110	13	PAK1_MOUSE	Serine/threonine-protein kinase PAK 1	51	3	61041	5.53	−1.3	0.017
115	16	DPYL2_MOUSE	Dihydropyrimidinase-related protein 2	617	15	62638	5.95	−1.1	0.022
527	33	GMFB_MOUSE	Glia maturation factor beta	137	42	16884	5.08	−1.4	0.023
30	17	ACON_MOUSE	Aconitate hydratase, mitochondrial	844	17	86151	8.08	−1.2	0.026
177	19	COR1A_MOUSE	Coronin-1A	234	11	51641	6.05	−1.3	0.028
113	18	GLSK_MOUSE	Glutaminase kidney isoform, mitochondrial	235	5	74771	8.23	−1.1	0.028
83	20	VATA_MOUSE	V-type proton ATPase catalytic subunit A	779	21	68625	5.42	−1.2	0.029
32	21	ACON_MOUSE	Aconitate hydratase, mitochondrial	933	17	86151	8.08	−1.2	0.03
90	24	ODP2_MOUSE	Dihydrolipoyllysine-residue acetyltransferase component of pyruvate dehydrogenase complex, mitochondrial	811	15	68469	8.81	−1.2	0.033
91	27	GRP75_MOUSE	Stress-70 protein, mitochondrial	213	6	73768	5.91	−1.2	0.038
160	28	TCPB_MOUSE	T-complex protein 1 subunit beta	88	4	57783	5.97	−1.1	0.04
448	30	HPRT_MOUSE	Hypoxanthine-guanine phosphoribosyltransferase	134	9	24783	6.21	−1.1	0.041
74	31	VATA_MOUSE	V-type proton ATPase catalytic subunit A	345	11	68625	5.42	−1.2	0.042
129	32	CH60_MOUSE	60 kDa heat shock protein, mitochondrial	714	17	61088	5.91	−1.2	0.05

a Protein ID assigned manually.

b Protein name identified by MS.

c Sequence coverage achieved by MALDI-TOF-MS.

d Theoretical molecular weight of the protein(s).

e Theoretical isoelectrical point of the protein(s).

f The ratio in spot density from EE-treated TRPC1^−/−^ mice compared to TRPC1^−/−^ mice. *n* = 6 for both group.

**Table 3:** Differentially expressed protein spots in between EE treated-WT mice and non-treated WT mice identified via 2D-DIGE/MALDI-TOF-MS/MS

Spot No	Protein ID. ^a^	Accession No.	Protein name ^b^	Mascot score	Cove. ^c^ (%)	MW^d^ (Da)	PI ^e^	WT+EE vs WT	
								Ratio^f^	*P*-value
93	1	ODP2_MOUSE	Dihydrolipoyllysine-residue acetyltransferase component of pyruvate dehydrogenase complex, mitochondrial	383	9	68469	8.81	−1.1	0.015
281	2	PRS7_MOUSE	26S protease regulatory subunit 7	124	4	49016	5.72	1.29	0.025
151	4	DPYL2_MOUSE	Dihydropyrimidinase-related protein 2	460	13	62638	5.95	1.27	0.04

a Protein ID assigned manually.

b Protein name identified by MS.

c Sequence coverage achieved by MALDI-TOF-MS.

d Theoretical molecular weight of the protein(s).

e Theoretical isoelectrical point of the protein(s).

f The ratio in spot density from EE-treated WT mice compared to WT mice. *n* = 6 for both group.

### Identification of differentially expressed proteins in hippocampus between WT and TRPC1^−/−^ mice

A total of 10 proteins were found to be differentially expressed between WT mice and TRPC1^−/−^ mice (Table 1). Among these proteins, 8 protein spots were up-regulated in hippocampus in TRPC1^−/−^ mice relative to the WT mice and 2 proteins were down-regulated. The significantly up-regulated proteins in hippocampus included phosphoglycerate mutase 1, serum albumin, voltage-dependent anion-selective channel protein 2 (VDAC 2), ATP synthase subunit alpha, mitochondrial, dihydropyrimidinase-related protein 2, serum albumin, far upstream element-binding protein 2, and v-type proton ATPase catalytic subunit A. The significantly down-regulated proteins included dihydrolipoyllysine-residue acetyltransferase component of pyruvate dehydrogenase complex, mitochondrial, and heme-binding protein 1.

The PANTHER classification system revealed that these proteins could be classified into several groups according to their functional properties. The most dominant functions of the identified proteins involved catalytic activity (38.5%) followed by transporter activity (23.1%), binding (23.1%) and receptor activity (15.4%). These proteins could be classified into 6 categories according to their biological processes, including metabolic process, localization, apoptosis process, cellular process, development process and multicellular organismal process. Among these differential proteins, far upstream element-binding protein 2 and VDAC 2 were involved in apoptosis process. Phosphoglycerate mutase 1, v-type proton ATPase catalytic subunit A and ATP synthase subunit alpha were involved in ATP synthesis and glycolysis. Serum albumin is an abundant plasma protein. Heme-binding protein 1 is a 189 amino acid intracellular tetrapyrrole-binding protein that assists in prevention of cellular toxicity by removing free porphyrinogens from the cell.

### The effects of EE treatment on the expression of hippocampal proteins in TRPC1^−/−^ mice

A total of 22 proteins were found to be differentially expressed between EE-treated TRPC1^−/−^ mice and non-treated TRPC1^−/−^ mice (Table 2). Among these proteins, 2 protein spots were up-regulated in EE-treated TRPC1^−/−^ mice relative to non-treated TRPC1^−/−^ mice and 20 protein spots were down-regulated. The significantly up-regulated proteins included glyceraldehyde-3-phosphate dehydrogenase and glyceraldehyde-3-phosphate dehydrogenase. The significantly down-regulated proteins included ATP synthase subunit beta, mitochondrial, dihydropyrimidinase-related protein 2, α-internexin, vesicle-fusing ATPase, beta-actin-like protein 2, dihydropyrimidinase-related protein 2, serine/threonine-protein kinase PAK 1, dihydrolipoyllysine-residue acetyltransferase component of pyruvate dehydrogenase complex, mitochondrial, aconitate hydratase, mitochondrial, coronin-1A, dihydropyrimidinase-related protein 2, v-type proton ATPase catalytic subunit A, glia maturation factor beta, glutaminase kidney isoform, mitochondrial, stress-70 protein, mitochondrial, aconitate hydratase, mitochondrial hypoxanthine-guanine phosphoribosyltransferase, t-complex protein 1 subunit beta, v-type proton ATPase catalytic subunit A, and 60 kDa heat shock protein, mitochondrial.

The PANTHER classification system revealed that these proteins could be classified into several groups according to their functional properties. The most dominant functions of the identified proteins involved catalytic activity (47.8%) followed by binding (21.7%), receptor activity (8.7%), transporter activity (8.7%), structural molecule (8.7%) and enzyme regulator activity (4.3%). These proteins could be classified into 8 categories according to the biological processes, including metabolic process, localization, cellular process, development process, multicellular organismal process, response to stimulus, cellular component organization or biogenesis and biological regulation. Among these proteins, α-internexin and GMF-β were two memory-associated proteins. 60 kDa heat shock protein was a pro-apoptotic protein. ATP synthase subunit beta, aconitate hydratase, vesicle-fusing ATPase, glutaminase kidney isoform and v-type proton ATPase catalytic subunit A were involved in ATP synthesis, the TCA cycle and glycolysis, respectively. Stress-70 protein was involved in oxidative stress. Glyceraldehyde-3-phosphate dehydrogenase was involved in metabolic processes. T-complex protein 1 subunit beta is a subunit of the hetero-oligomeric complex CCT (chaperonin containing TCP-1) present in the eukaryotic cytosol. Coronin-1A is preferentially expressed in hematopoietic cells, and it carries a coiled-coil domain of the leucine zipper variety which mediates the formation of homotrimeric complexes [29]. The dihydropyrimidinase related protein 2 (DRP-2) plays a vital role in the axonal growth and guidance [30].

### Identification of differentially expressed proteins in hippocampus between EE-treated WT mice and non-treated WT mice

A total of 3 protein spots were found to be differentially expressed between EE-treated WT mice and non-treated WT mice (Table 3). Among these proteins, 2 protein spots were up-regulated in EE-treated WT mice relative to WT mice and 1 protein spots were down-regulated. The significantly up-regulated hippocampal proteins included 26S protease regulatory subunit 7 and dihydropyrimidinase-related protein 2. The significantly down-regulated proteins was dihydrolipoyllysine-residue acetyltransferase component of pyruvate dehydrogenase complex, mitochondrial.

PANTHER analysis revealed that these proteins were involved in catalytic activity in metabolic process according to their functional properties, suggesting that EE treatment may exert effects on metabolism in hippocampus in WT mice.

### Validation of differentially expressed proteins by western-blot analysis

**Figure 7:**
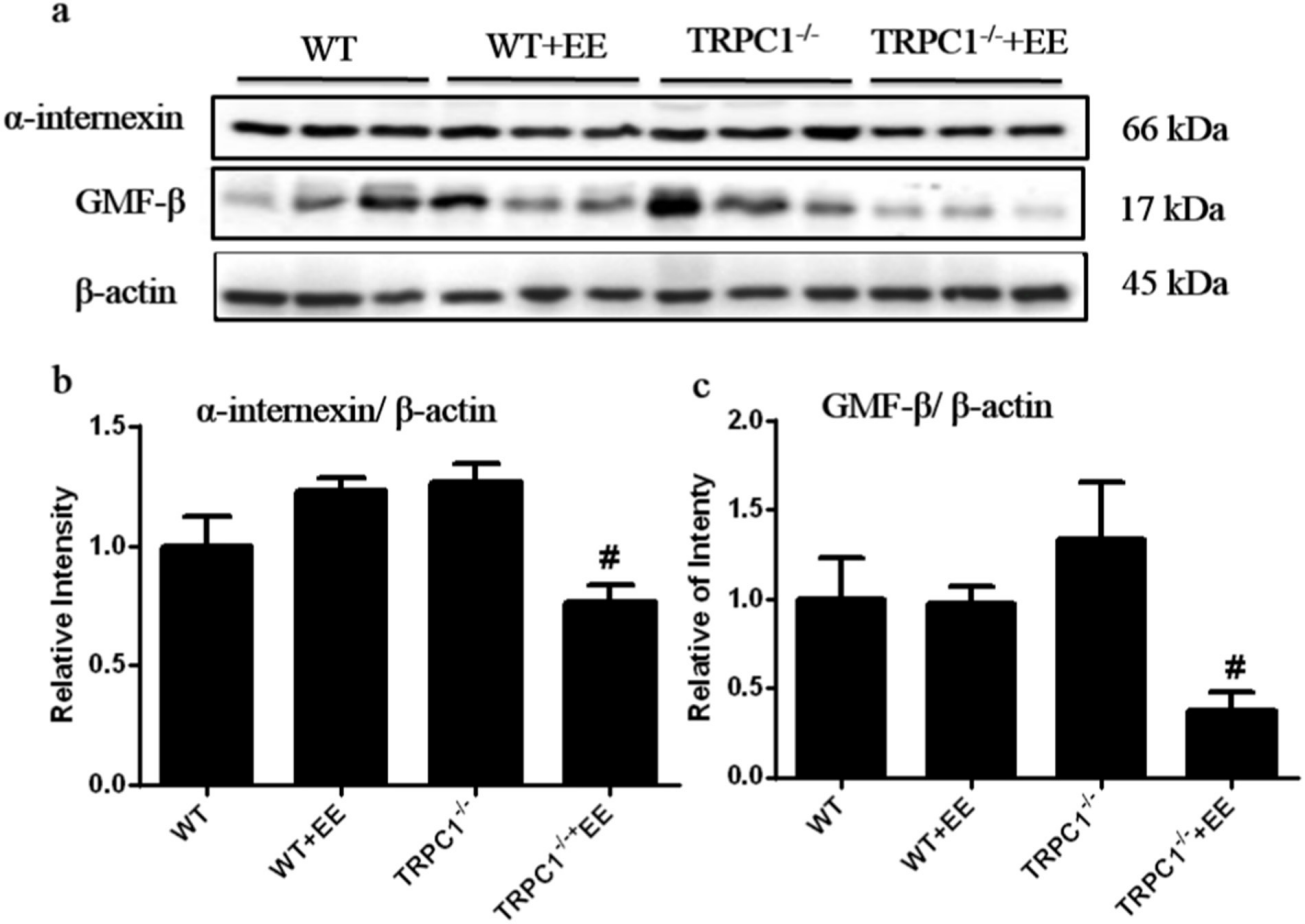
Verification of α-internexin and GMF-β by Western-blot analysis **a** and **b.** The relative levels of α-internexin in hippocampus in WT mice and TRPC1^−/−^ mice treated with or without EE. **a** and **c.** The relative levels of GMF-β in WT mice and TRPC1^−/−^ mice treated with or without EE. ^*^P < 0.05 versus WT mice, ^#^*P* < 0.05 versus TRPC1^−/−^ mice. *n* = 3 for each group. Values were expressed as mean +/− SEM.

To confirm the data by 2D-DIGE, Western-blot analysis was performed. Among the differentially expressed proteins, two memory-related molecules, α-internexin and GMF-β, were selected for validation. A trend of up-regulation of both α-internexin and GMF-β was shown in hippocampus in TRPC1^−/−^ mice compared to the WT mice (Figure 7a–7c), while the treatment of EE significantly decreased the expression of both α-internexin and GMF-β in TRPC1^−/−^ mice (Figure 7a–7c). These data further validated the data by 2D-DIGE.

### TRPC1 depletion caused neuronal loss and apoptosis in hippocampus

**Figure 8:**
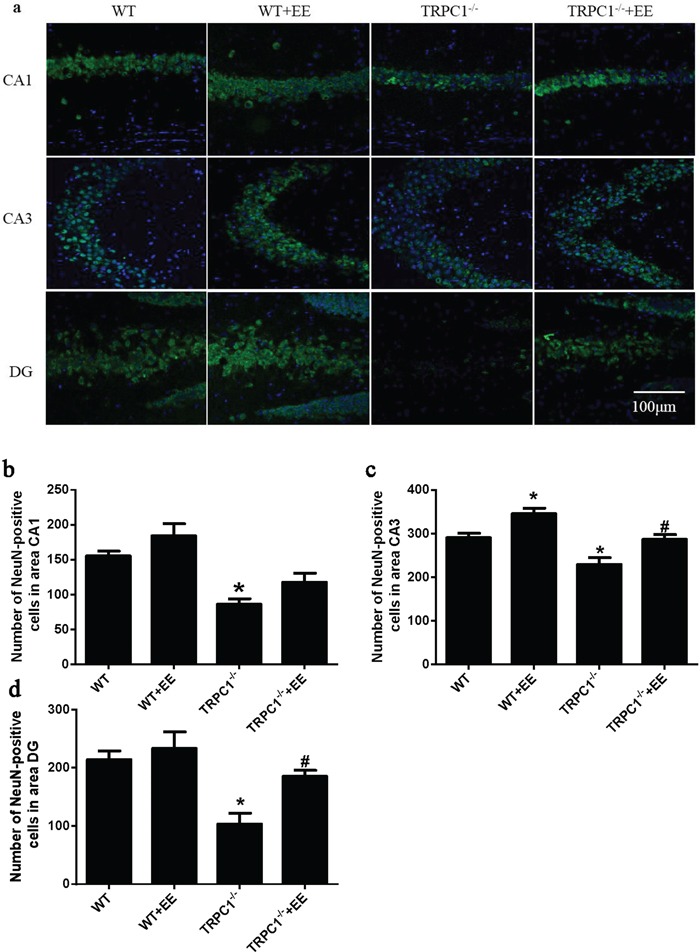
TRPC1 depletion caused neuronal loss **a.** Immunofluorescent images depicting the number of neurons as evidenced by NeuN-staining. **b.** The number of NeuN-positive cells in CA1. **c.** The number of NeuN-positive cells in CA3. **d.** The number of NeuN-positive cells in DG.^*^ and ^**^P < 0.05 and *P* < 0.01 *vs* WT mice, respectively; ^#^ and ^##^ *P* < 0.05 and *P* < 0.01 *vs* TRPC1^−/−^ mice, respectively. All the values were expressed as mean +/− SEM. *n*=3 for each group. Scale bar = 100 μm.

**Figure 9:**
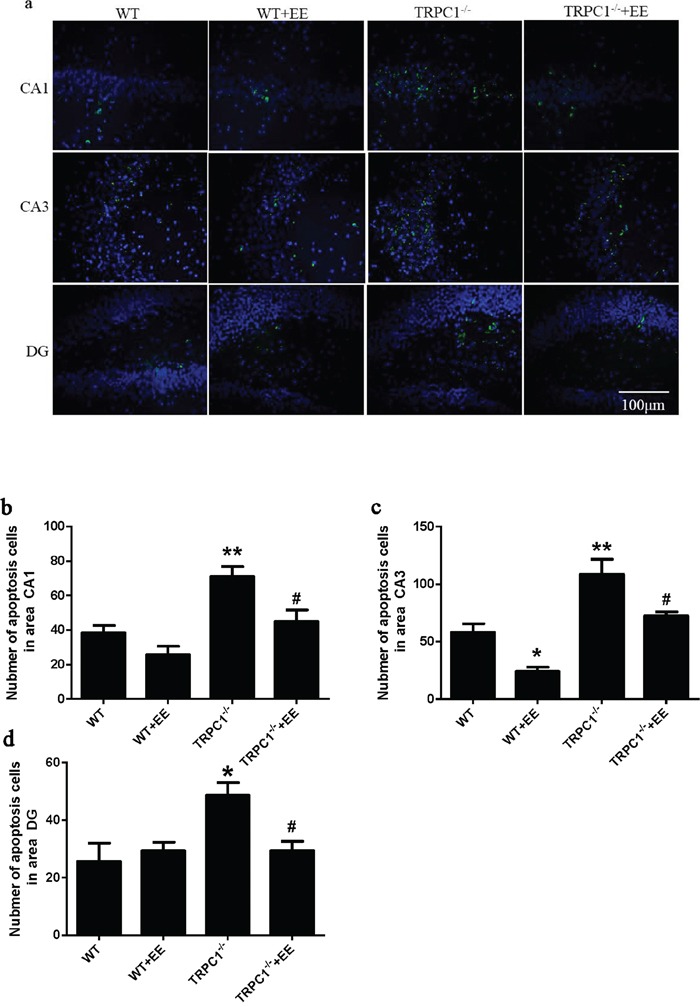
TRPC1 depletion caused hippocampal neuronal apoptosis **a.** A representative image of hippocampal neuronal apoptosis. **b.** The number of apoptotic cells in CA1. **c.** The number of apoptotic cells in CA3. **d.** The number of apoptotic cells in DG.^*^ and ^**^P < 0.05 and *P* < 0.01 *vs* WT mice, respectively; ^#^ and ^##^*P* < 0.05 and *P* < 0.01 *vs* TRPC1^−/−^ mice, respectively. All the values were expressed as mean +/− SEM. *n*=3 for each group. Scale bar = 100 μm.

In order to determine the effects of TRPC1 depletion on neuronal survival, we performed immunofluorescence staining using neuron-specific marker NeuN and TUNEL staining to evaluate the changes of apoptosis in hippocampus. The data showed the number of NeuN-positive cells was significantly decreased in hippocampal CA1, CA3 and dentate gyrus (DG) of TRPC1^−/−^ mice compared to the WT mice (*P* < 0.05) (Figure 8a–8d), while the treatment of EE significantly increased the number of NeuN-positive cells in TRPC1^−/−^ mice (*P* < 0.05) (Figure 8a–8d). The data showed TUNEL-positive cells were significantly increased in hippocampal CA1, CA3 and DG of TRPC1^−/−^ mice relative to the WT mice (*P* < 0.05) (Figure 9a–9d). TUNEL-positive cells were significantly decreased in hippocampus of TRPC1^−/−^ mice treated with EE compared with non-treated TRPC1^−/−^ mice (*P* < 0.05) (Figure 9a–9d). These data suggested that TRPC1 depletion caused neuronal loss and apoptosis, and EE treatment could prevented neuronal loss and apoptosis in hippocampus of TRPC1^−/−^ mice.

## DISCUSSION

TRPC1 is highly expressed in hippocampus and involved in the regulation of proliferation, indicating that TRPC1 may be involved in the process of spatial memory [6, 13]. EE was shown to modulate hippocampal-dependent behavior in rodents [31]. Our study demonstrated that TRPC1 depletion caused proteomic change in hippocampus and spatial memory impairment, and EE could rescue memory impairment caused by TRPC1 depletion.

The behavioral tests, i.e. the trace cued and contextual fear conditioning, Y maze test and step-down test, consistently demonstrated that TRPC1 depletion led to spatial memory impairment in mice. The proteomic data suggested that TRPC1 regulates directly or indirectly the expression of a number of proteins in hippocampus. These proteins were involved in metabolic process, localization, apoptosis process, cellular process, development process and multicellular organismal process. However, neuronal apoptosis could cause memory impairment especially in regard to the hippocampus [32]. Voltage-dependent anion-selective channel protein 2 (VDAC 2) and far upstream element-binding protein 2 were apoptosis-related proteins. VDAC 2 was a channel associated with cellular apoptosis. The recombinant pro-apoptotic proteins Bax and Bak were able to accelerate the opening of VDAC, whereas the anti-apoptotic protein Bcl-xL closes VDAC by binding to it directly [33]. In the present study, the expression level of VDAC 2 was significantly higher in TRPC11^−/−^ mice compared to WT mice, and the up-regulation was prevented by EE treatment in TRPC1^−/−^ mice, suggested VDAC 2 could be involved in apoptosis, in turn, memory impairment caused by TRPC1 depletion. Based on the functional analysis on differentially expressed proteins in hippocampus, we speculated that neuronal apoptosis-related molecule VDAC 2 could be involved in spatial memory impairment in TRPC1^−/−^ mice. However, the specific roles of this protein in memory deficit in TRPC1^−/−^ remain to be further elucidated.

EE was able to facilitate enhanced sensory, cognitive and motor stimulation of mice [14, 15], and was shown to enhance learning and memory [34, 35] and the mechanisms may involve hippocampal neurogenesis [15]. In this study, we further demonstrated that EE could rescue spatial memory impairment caused by TRPC1 depletion as measured by three behavioral tests. Furthermore, we also revealed that EE could modulate the expression of a number of proteins in hippocampus in TRPC1^−/−^ mice. In order to better understand the possible involvement of these differentially expressed proteins in amelioration of memory impairment in TRPC1^−/−^ mice by EE, in the following section, we focused on the discussion of differentially expressed proteins associated with memory and apoptosis.

Alpha-internexin is a constitutional intermediate neurofilament protein first described in 1985 [36]. A growing body of evidence has demonstrated that α-internexin is involved in neurodegenerative diseases associated with neurofilament accumulation and mislocation [37]. Transgenic mice overexpressing α-internexin proteins displayed dis-organization of the IF network, which could cause neurofilament mis-accumulation, progressive neurodegeneration, and loss of neurons ultimately [38]. Furthermore, overexpression of α-internexin in PC12 cells induced apoptosis-like cell death [39]. Our data showed a trend of up-regulation of α-internexin in hippocampus in TRPC1^−/−^ mice and EE could significantly suppress the expression of α-internexin, indicating an involvement of α-internexin in memory impairment in TRPC1^−/−^ mice and the protective effects of EE on memory.

GMF was shown to activate a number of genes related to pro-inflammatory effects in the nervous system, such as tumor necrosis factor-α (TNF-α), interleukin 1-beta (IL-1β), 12-lipoxygenase and chemokine CX3C, which were involved in the pathophysiology of neurodegenerative disorders such as AD [40]. It was reported that the overexpression of GMF in astrocytes led to the destruction of primary oligodendrocytes and neurons by interactions between highly purified cultures of astrocytes, microglia, oligodendrocytes and neurons [41]. Besides, it was demonstrated that spatial memory retention was improved in GMF knockout mice compared to WT controls following Aβ infusion [40]. These data indicated crucial roles of GMF in pro-inflammatory responses and memory. In this study, we demonstrated a trend of up-regulation of GMF-β in hippocampus in TRPC1^−/−^ mice and EE could significantly down-regulate the expression of GMF-β, indicating an involvement of GMF-β in memory impairment in TRPC1^−/−^ mice and the protective effects of EE against spatial memory deficit as observed.

Heat shock protein-60 was a pro-apoptotic protein, which can activate caspase 3 and thus leads to cellular apoptosis [42]. Our present study showed that EE could down-regulate the expression of heat shock protein-60 in hippocampus of TRPC1^−/−^ mice, suggesting that the protective effects of EE against memory deficit in TRPC1^−/−^ mice may also involve the down-regulation of Heat shock protein-60.

The dihydropyrimidinase related protein 2 (DRP-2) is involved in the axonal growth and guidance, and its expression was increased in AD brains, suggesting a role in impaired neural network formation in AD [30]. In the present study, we found that the expression of DRP-2 was significantly up-regulated in hippocampus in TRPC1^−/−^ mice compared to WT mice, and EE treatment prevented the up-regulation of DRP-2 in hippocampus in TRPC1^−/−^ mice. It was suggested that dys-regulation of DRP-2 in hippocampus may lead to impairment of the brain functions such as spatial memory ability, and EE treatment could rescue the impairment via modulating the expression of DRP-2, as evidenced by modulation of DRP-2 expression by EE treatment in TRPC1^−/−^ mice.

In summary, our current data demonstrated that TRPC1 depletion altered the expression of a number of proteins in hippocampus and spatial memory impairment, and that EE treatment could rescue memory deficit caused by TRPC1 depletion. These data indicated that TRPC1 regulated directly or indirectly the expression of multiple proteins, which could be crucial for the maintenance of spatial memory ability, and that EE treatment could prevent spatial memory impairment through its modulation effects on the expression of those dys-regulated proteins such as α-internexin and GMF-β in hippocampus.

## MATERIALS AND METHODS

### Animals and treatment protocol

The TRPC1^−/−^ mice were supplied by Prof. Lutz Birnbaumer (NIEHS, US) and the control mice were purchased from Vital River Laboratory Animal Technology Co. Ltd (Beijing, China). All the animal experiments were performed according to the “Policies on the Use of Animals and Humans in Neuroscience Research” revised and approved by the Society for Neuroscience in 1995, and all the efforts were made to minimize animal suffering to reduce the number of animals used. The mice were kept in cages under a 12-hour light/12-hour dark cycle with the light on from 7: 00 am to 7:00 pm at stable temperature (23–25°C) and humidity. All the mice were housed in groups of 10 mice per cage (470×350×200 mm) with free access to food and water.

A total of sixty mice at 3 months old were used in this study. The WT mice and TRPC1^−/−^ mice were randomly divided into four groups: WT mice, EE-treated WT mice, TRPC1^−/−^ mice and EE-treated TRPC1^−/−^ mice. The WT and TRPC1^−/−^ mice were treated by EE for 6 weeks.

### Trace cued and contextual fear conditioning

Trace cued and contextual fear memory is hippocampus-dependent memory [43]. Contextual fear conditioning consists of an initially neutral conditioned stimulus (CS), typically an auditory tone and an unconditioned stimulus (US), typically a foot shock. In the training session (Day 1), the mice were placed in white box and left to explore for 3 min. Tone (20 sec, 75 dB, 2800 Hz) and foot shock (2 sec, 0.5 mA) were separated by a 20-sec trace interval. All the mice received 5 tone-shock trials and a 200-sec inter-trial interval between CS onset and the next CS onset. After the last shock, the mice were left for 2 min. The mice were trained in the Freeze Monitor environment. Following the training session, all the mice were returned to their home cages. Urine and feces were cleaned with 75% ethanol after each test session to reduce the possibility of odor interference.

The auditory cued fear test sessions were performed on day 2, and 24 h after the training, and the mice were placed in black box instead of the white box. In order to change the smell of the environment, some orange peels were put in the black box. The mice was allowed freely to explore 2 min. The tone was delivered in the same way as on Day 1 during the training, but without foot shock. On Day 3, 48 h after the training, the mice were returned to the original training room for 60 min before being placed back in white box without CS or US for an 8 min context test.

### The recognition Y-maze test

Y-maze is a Y-shaped apparatus, with three arms (start arm, novel arm and other arm), each 44 cm long and 15 cm wide, with walls 10 cm high. The arms are at a 120° angle from each other. Three arms were randomly designated: start arm, novel arm, and other arm. The start arm and other arm were randomly designed to avoid the spatial memory error. Urine and feces were cleaned with 75% ethanol after each test session to reduce the possibility of odor interference.

The Y-maze test consisted of 2 trials separated by an inter-trial interval (ITI). During the training phase, the novel arms were blocked off, and the mice were allowed to explore the start arm and the other arm for 10 min. 60 min later, the 2nd trial (retention) was conducted. For the 2nd trial, the mice were placed back in the Y-maze for 5 min, with all three arms open. Using a ceiling-mounted CCD camera, all the trials were recorded on a VCR. Video recordings were later analyzed and the number of entries, the time spent and the distance traveled in each arm were analyzed. Because of their natural curiosity, the mice prefer to explore the novel arm. We define the ratio of entry as the total entries into the novel arm versus the total entries into any arm of the maze, the ratio of time as the total time spent in the novel arm versus the total time spent in any arm of the maze, the ratio of distance as the total distance in the novel arm versus the total distance in any arm of the maze. These parameters are indicative of spatial recognition memory of the mice.

### Step-down test

The training apparatus was a 60 cm × 10 cm× 10 cm plastic box, the floor of which was made of parallel 0.1 cm-caliber stainless steel bars spaced 0.5 cm apart. An elevated rubber platform (diameter: 10 cm, height: 4.5 cm) was placed in the middle of the training box apparatus. On the first training day, the mice were first exposed to a 5 min learning course, during which they were placed on the platform, and then energized (36 V, AC) for 5 min. If the animals stepped down from the platform (“error trial”), they were punished by an electric foot shock. The numbers of “errors” (steps down from the platform) and the time for mice to jump on the platform for the first time during the training period were recorded. After 24 h, mice were placed on the platform, and the numbers of “errors” and the time for mice to steps down from the platform for the first time were also recorded, which was taken as a measure of memory retention.

### Sample preparation for 2-D electrophoresis

The mice treated with 6 weeks were sacrificed after behavioral tests. The hippocampi were isolated on the ice and then stored at −80°C. The brain samples were suspended in DIGE-specific lysis buffer (7M urea, 2M thiourea, 30 mM Tris-HCl, 4% CHAPS, pH 8.5) and ultrasonicated for 2 min in cycles of 3s on and 5 s off at 35% power using a Fisher 550 Sonic Dismembrator (Pittsburgh, PA, USA) until the samples are clarified. The samples were incubated on the ice for 30 min, and then centrifuged subsequently at 20,000 g at 4°C for 60 min. The supernatants were ultrafiltered at 14,000 g at 4°C for 30 min to remove salt and other impurities, and then resuspended in DIGE-specific lysis buffer. The protein solutions were collected and stored at −80°C. The protein concentrations were determined by 2-D Quant Kit (GE health care, USA) in accordance to the manufacturer's protocol.

### DIGE labeling of protein samples

Each CyDye stock was reconstituted in 99.8% anhydrous N, N-Dimethylformamide (DMF, Sigma 227056) to give a final dye concentration of 1 nmol/μL. A working solution of 200 pmol/μL of each CyDye used to label proteins were generated by dilution of stock solutions with DMF. All the samples from WT and TRPC1^−/−^ mice were diluted to 5 μg/μL after the protein quantification. A pooled internal standard was composed of 5 μL of all the samples from WT and TRPC1^−/−^ mice. A dye swap was performed to control for preferential labeling by one of the dyes or for the different fluorescent characteristics of the gel at the different wavelengths. 5 μ of each protein sample (25 μg) with a pH of 8.0–9.0 was minimally labeled with 200 pmol Cy3 (GE Healthcare, 25-8008-61) or Cy5 (GE Healthcare, 25-8008-62), and the pooled internal standard was labeled with Cy2 (GE Healthcare, 25-8008-62). The labeling reaction was incubated on ice in the dark for 30 min. The reaction was quenched by addition of 10 mM lysine (Sigma, L5626) for 10 min at 4°C in the dark. After the protein samples were labeled, the Cy2-, Cy3- and Cy5-labeled samples were mixed together and 80 μL of 2×lysis buffer (8 M urea, 2% CHAPS, 0.2% DTT, 2% (v/v) IPG buffer, pH 3-11 NL, 0.002% bromophenol blue) was added to each mixture and incubated on ice for 10 min in the dark. Rehydration buffer was then added to make the total volume of the sample up to 450 μL.

### 2-D Difference gel electrophoresis (2D-DIGE)

The first dimension was performed on Ettan IPGphor Isoelectric Focusing System (GE Healthcare). A total 75 μg of each labeled samples were put into 24 cm pH 3-11 NL Immobiline DryStrips (GE Healthcare). Then 2 mL of mineral oil was added to cover each strip to reduce solvent evaporation. Proteins were taken up into strips by active rehydration at 50 V for 18 h. Isoelectric focusing (IEF) conditions were step 300 V for 12 h, step 500V for 2 h, step 1000V for 2 h, gradient 8000 V for 8 h, step 8000V for 8 h at 20°C and the temperatures of the room was kept at 18°C. Following isoelectric focusing, each strip was equilibrated with a reducing equilibration buffer composed of 6 M urea, 75 mM Tris-HCl buffer (pH 8.8), 30% (v/v) glycerol, 2% (w/v) SDS, and 1% (w/v) DTT for 15 min at room temperature on the shaking table. Subsequently, strips were re-equilibration in the same buffer containing 6 M urea, 75 mM Tris-HCl buffer (pH 8.8), 30% (v/v) glycerol, 2% (w/v) SDS, and 4.5% (w/v) IAA. The equilibrated strips were loaded on the top of 12.5% SDS-PAGE gels with 0.5% (w/v) ultralow melting point agarose sealing solution (25 mM Tris, 192 mM glycine, 0.1% SDS, 0.5% (w/v) agarose, 0.002% (w/v) bromophenol blue). Electrophoresis was performed using an Ettan DALTsix Electrophoresis System (GE Healthcare) and using the following conditions: 1 W/gel for 1 h, then 10 W/gel for 6 h at 15°C in the dark. Following 2-D electrophoresis of the DIGE gels, they were immediately scanned using a Typhoon TRIO Variable Mode Imager (GE Healthcare). Images scanned at 100 μm resolution on the Typhoon scanner were cropped to an appropriate field of interest in Image Quant. To control for variation in the signal across gels, the PMT was set to ensure that the maximum pixel intensity of all of the gel images remained within a range of 40,000–60,000 pixels.

### Image analysis

The DIGE gels in this experiment were analyzed using the DeCyder software package (Version 6.5 GE Healthcare) to following the manufacturer's protocol. Following confirmation of appropriate spot detection, matching, and normalization, spot statistics were reviewed. Both DeCyder and Progenesis incorporated the use of a Student's *t* test to quantify differential expression of spots between the experimental groups. The normalized volume of a spot is compared across the gels between the replicate groups and spots that were found to be statistically significant (*p*≤0.05) were isolated for further investigation. The differentially expressed protein spots were identified by mass spectrometry.

### Spot picking and in-gel digestion

A total of 1 mg of hippocampal protein was used to run 2-DE using identical conditions as above. The gel was stained with coomassie blue solution (0.12% Coomassie Brilliant Blue G-250, 10% phosphoric acid, 20% ethanol, 10% ammonia sulfate). The protein spots exhibiting significant changes (*p*≤0.05) detected by Decyder software analysis were manually excised from the Coomassie brilliant bluestained gel and destained with 50% acetonitrile in 25 mM ammonium bicarbonate followed by dehydration in 100% acetonitrile. After the reagents were removed, the gel pieces were digested with 0.15 μg of sequencing-grade trypsin (Promega, Madison, Wisconsin, USA) in 15 μL digestion buffer containing 25 mM ammonium bicarbonate. The mixture was incubated overnight at 37°C, then used to mass spectrometry.

### Mass spectrometry

Protein identification was carried out on AB SCIEX MALDI-TOF/TOF 5800 mass spectrometry (Foster City, CA, USA). A total of 0.6uL of peptide extract was used for the MADL-TOF-MS analysis and crystallized with 0.6 μL 10 mg/mL α-cyano-4-hydroxycinnamic acid (CHCA) in 0.1% TFA, 50% acetonitrile (ACN) directly on the target and dried at room temperature. The spectra were externally calibrated. MASCOT was used for database searching against the SwissProt databases (Matrix Science, UK) for the mice brain proteins. The search was carried out in Mus musculus database and conducted with a tolerance on mass measurement of 100 ppm in MS mode and 0.5 Da in MS/MS mode. Up to two missed cleavage per peptide was allowed. A fixed carbamidomethyl modification was taken into account.

### Western-blot analysis

To confirm the data by 2D-DIGE, the expression of two proteins was further measured by Western-blot analysis. Hippocampal proteins from WT mice, TRPC1^−/−^ mice with or without EE treatment were extracted by using lysis buffer (Beyotime, China) with protease and phosphatase inhibitor cocktail (Thermo Scientific, USA). The concentration of total proteins was measured by BCA protein assay kit (Thermo Scientific, USA). Protein samples were mixed with loading buffer (10% v/v) and heated at 100°C for 5 min, then separated on 12% PAGE gels with 4% stacking gels and transferred to PVDF membranes. Membranes containing the transferred proteins were blocked with 5% non-fat milk in TBST (150 mM NaCl, 10 mM Tris, 0.1% Tween-20, pH 8.0). Then membranes were incubated with anti-α-internexin (1: 10000, Abcam, ab40758), anti-GMF-β (1:1000, Santa cruz, sc-134347) and anti-β-actin (1:1000, Santa cruz, sc-47778) in TBST buffer overnight on ice on the shaking table. After washing in TBST (4 × 10 min), the membranes were incubated with a 1:3000 dilution of anti-rabbit or anti-mouse IgG HRP secondary antibody diluted in TBST for 1 h. Then the membranes were washed in TBST (4×10 min) and developed using chemiluminiscence reagents from an ECL kit (Pierce). Blots were detected on a phosphorimager and analyzed using ImageQuant 1D software.

### Immunofluorescence staining and TUNEL assay

Mouse brain tissues were fixed in 4% paraformaldehyde for two days and dehydrated in ethanol and embedded in paraffin. The coronal mouse brain sections were sectioned. After dewaxing and rehydration, the sections were treated with 0.01M citrate buffer (pH 6.0) with 0.1% Tween-20 at 95–100°C for 5 min for antigen retrieval. The sections were incubated at 4°C overnight with the primary antibody (NeuN at 1 : 5000). After washing with PBS, the sections were stained with secondary antibody, Alexa Fluor^®^-488 for 1 h in the dark, and then stained with DAPI (Beyotime Institute of Biotechnology, Haimen, China) for 1 min to reveal the nuclei. The sections were examined with a laser scanning confocal microscope. For the measurement of apoptosis, TUNEL assay was performed using the DeadEnd™ Fluorometric TUNEL System as described in the instruction provided by the kit. The images were taken using a microscope (Olympus 1X51, Tokyo, Japan).

### Statistical analysis

The data were expressed as mean +/− standard error (SEM) and analyzed using SPSS 18.0 statistical software (SPSS Inc. Chicago, Illinois, USA). One-way ANOVA was used to determine the different means among the groups. The level of significance was set at *P* < 0.05.

## SUPPLEMENTARY FIGURES


