# Supplementary material for: Spatial memory impairment by TRPC1 depletion is ameliorated by environmental enrichment
Source: Oncotarget. 2016 Mar 28;7(19):27855–73. doi: 10.18632/oncotarget.8428 (PMC5053693; doi:10.18632/oncotarget.8428)
Supplement: Supplementary file 1 [file oncotarget-07-27855-s001.pdf]

SUPPLEMENTARY FIGURES

a

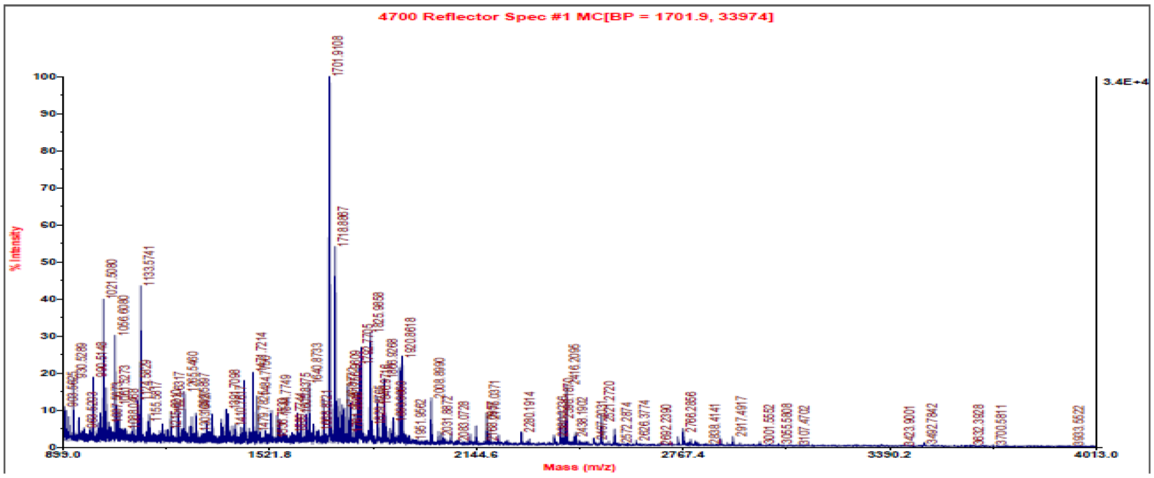

b

```
Sequence Coverage: 12%

Matched peptides shown in Bold Red

  1 MSFGSEHYLC SASSYRKVFG DSSRLSARLS GPGGSGSFRS QSLSRSNVAS
 51 TAACSSASSL GLGLAYRRLP ASDGLDLSQA AARTNEYKII RTNEKEQLQG
101 LNDRFAVFIE KVHQLETQNR ALEAELAALR QRHAEPSRVG ELFQRELREL
151 RAQLEEASSA RAQALLERDG LAEEVQRLRA RCEEESRGRE GAERALKAQQ
201 RDVDGATLAR LDLEKKVESL LDELAFVRQV HDEEVAELLA TLQASSQAAA
251 EVDVAVAKPD LTSALREIRA QYESLAAKNL QSAEEWYKSK FANLNEQAAR
301 STEAIRASRE EIHEYRRQLQ ARTIEIEGLR GANESLERQI LELEERHSAE
351 VAGYQDSIGQ LESDLRNTKS EMARHLREYQ DLLNVKMALD IEIAAYRKLL
401 EGEETRFSTG GLSISGLNPL PNPSYLLPPR ILSSTASKVS SAGLSLKKED
451 EEEEEEEEDA SKEVSKKTSK VGEGFEETLG EAVISTKKTG KSATEESTSS
501 SQKM
```

c

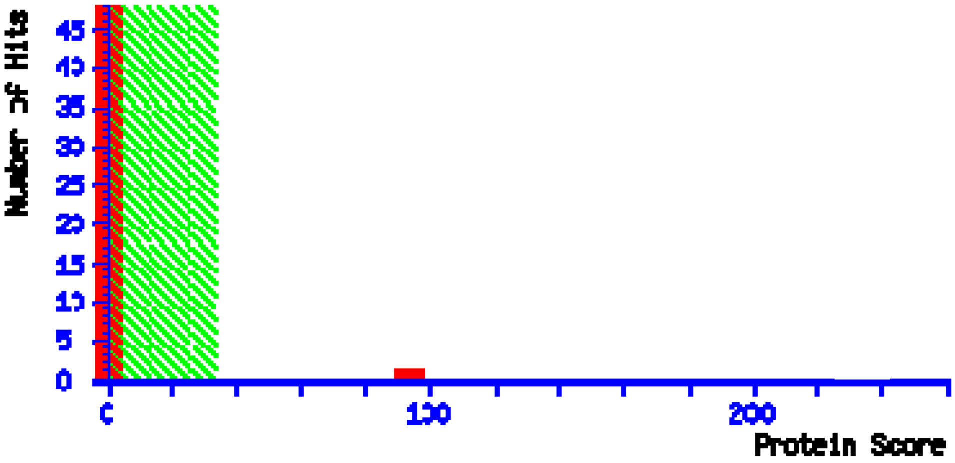

Supplementary Figure S1: Identification of α-internexin. a. The MALDI-TOF-MS map of α-internexin; **b.** The amino acid sequences of α-internexin in which matched peptide sequence was in red, and the sequence coverage was 12%; **c.** Protein score.

a

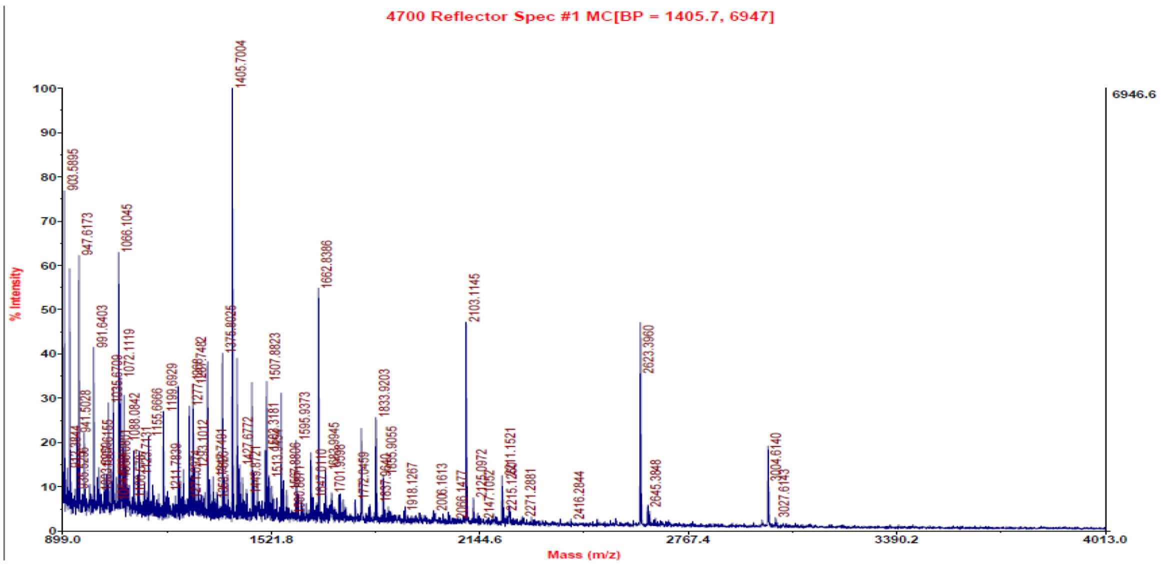

b

Sequence Coverage: 42%

Matched peptides shown in Bold Red

1 M**SESLVVCDV AEDLVEKLR**K FRFRKETHNA AIIMKIDKDE R**LVVLDEELE**
51 **GVSPDELKDE LPER**QPR**FIV YSYK**YQHDDG RVSYPLCFIF SSPVGCKPEQ
101 QMMYAGSKNK LVQTAELTKV FEIR**NTEDLT EEWLREK**LGF FH

c

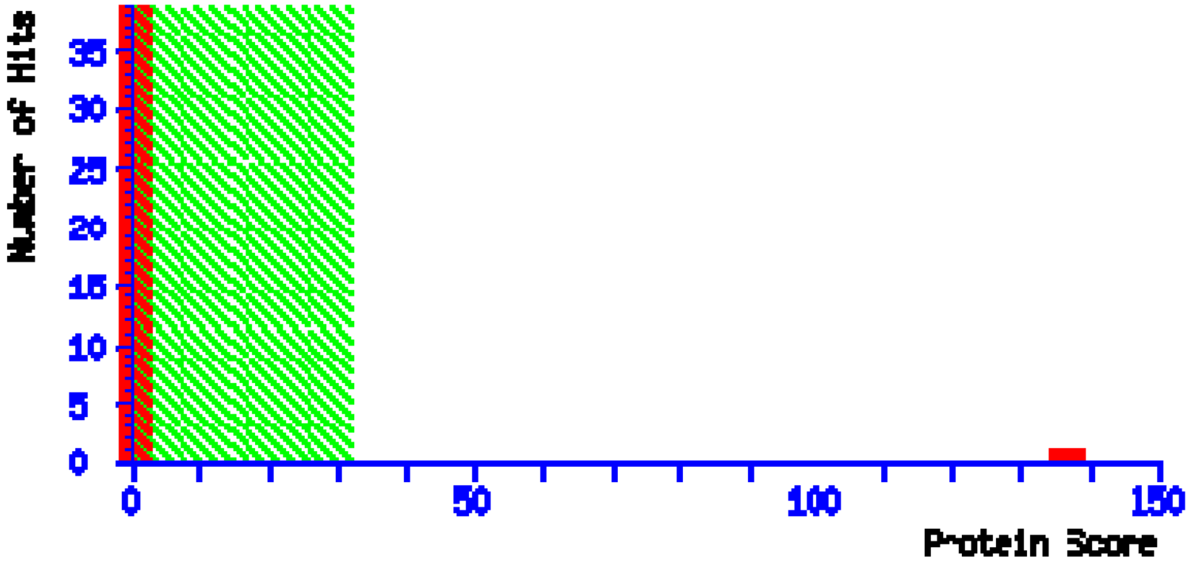

Supplementary Figure S2: Identification of GMF-β. a. The MALDI-TOF-MS map of GMF-β; **b.** The amino acid sequences of GMF-β in which matched peptide sequence was in red, and the sequence coverage was 42%; **c.** Protein score.
